# Therapeutic effects of Pulpotomy and Pulpectomy on deciduous molars with deep caries

**DOI:** 10.12669/pjms.336.13488

**Published:** 2017

**Authors:** Yuxiang Tang, Wantian Xu

**Affiliations:** 1 Yuxiang Tang, Department of Endodontics, Nanjing Stomatological Hospital, Medical School of Nanjing University, Central Road No. 30, Xuan Wu District, Nanjing 210008, China; 2 Wantian Xu, Department of Endodontics, Nanjing Stomatological Hospital, Medical School of Nanjing University, Central Road No. 30, Xuan Wu District, Nanjing 210008, China

**Keywords:** Deciduous molar, Deep caries, Pulpectomy, Pulpotomy

## Abstract

**Objective:**

To evaluate the therapeutic effects of pulpotomy and pulpectomy on deciduous molars with deep caries.

**Methods:**

A total of 124 children (192 molars) with deep caries treated from February 2014 to February 2015 were selected. They each had at least one molar with deep caries. MTA pulpotomy (101 molars) and Vitapex pulpectomy (91 molars) as well as prefabricated metal crown repair were conducted. The patients were followed up for 18 months after surgery, and the therapeutic effects were evaluated through clinical and X-ray examinations.

**Results:**

The proportion of molars without lesions was 80.20% in pulpotomy group, which significantly exceeded that of pulpectomy group (72.53%). The pulpotomy group with good clinical manifestations underwent spontaneous pain in four molars during follow-up, and five molars gradually underwent pain and gingival redness and swelling. The pulpectomy group suffered from occlusion discomfort in nine molars and gingival fistula in seven molars during follow-up. The postoperative morbidity of pulpectomy group was significantly higher than that of pulpotomy group (χ^2^=4.50, P=0.04). The 18-month tooth survival rates of pulpotomy and pulpectomy groups were 90% and 79% respectively, which were significantly different (χ^2^=4.645, P=0.031).

**Conclusion:**

The postoperative outcomes of pulpotomy are superior to those of pulpectomy.

## INTRODUCTION

Deciduous teeth are an important chewing organ during childhood^1^, playing a crucial role in the normal eruption of permanent teeth, normal jaw development and general health of children.^2^ The pulp exposure of deciduous teeth caused by dental caries or trauma and secondary inflammation affect local occlusal development, and even systemic physical or mental health.^3^ Therefore, it is critical to protect deciduous teeth and to improve the treatment of deciduous pulp. Currently, the prevalence of dental caries in children is as high as 50%-60%. Impaction pain and deep caries with largely damaged dental crown are commonly found upon first visit.^4^ Pulpotomy is mainly used for any deciduous teeth and young permanent teeth with vital pulp exposure, with a clinical success rate of 83%-100%. Pulpectomy is mainly applied to the teeth without necrosis in the root canal, such as various types of pulpitis and accidental pulp exposure, and the highest clinical success rate can reach up to 98%. However, due to drug characteristics and the infection degree of teeth, postoperative complications such as pulpitis, periapical inflammation and internal root canal absorption often occur.

The aim of this study was to assess the effects of pulpotomy and pulpectomy on deeply carious deciduous molars, and to observe the incidence of complications by clinical and X-ray examinations. The findings provide a valuable basis for the improvement of clinical treatment.

## METHODS

### Baseline clinical data

A total of 124 children (192 molars) with deep caries treated in our hospital from February 2014 to February 2015 were selected. This study has been approved by the ethics committee of our hospital. Before treatment, parents of the children were informed of the treatment process, possible risks, complications and necessity for dental pulp treatment. The parents have signed informed consent.

### Inclusion criteria

Healthy children aged 3-8 years old who could receive dental treatment under general anesthesia; deciduous molars with deep caries or those close to the site of removed pulp detritus; clinical and imaging examinations revealed deeply carious deciduous molars without pulpal degeneration (without spontaneous pain history, palpation pain, percussion pain, swelling, fistula, pathological movement or alveolar bone destruction at the root tip); available for prefabricated metal crown (PMC) repair after treatment.

### Exclusion criteria

Children with hereditary short tooth, root or early absorption of root, with drug allergy during treatment with poor compliance (e.g. unable to sign informed consent or to determine subsequent visit on time); with pulp disease, periapical diseases (e.g. spontaneous pain history and internal root canal absorption) or trauma history.

### Treatment methods

The enrolled patients were randomly divided into a pulpotomy group and a pulpectomy group. All the above examinations and treatments were performed by three dentists with over 5 years of experience in clinical practice.

### Pulpotomy group

1. X-ray examination was performed before surgery to explore periapical tissue and root conditions. There was no periodontal inflammation or absorption of the root which was in a stable state.
2. The surgical area was isolated under local anesthesia with primacaine, and saliva contamination was prevented with a suction device.
3. The surgical area was disinfected to prepare a cavity.
4. The cavity was flushed, its floor periphery was drilled using a disinfected dental bit, the roof of pulp chamber was removed, and the coronal pulp was dug out with a sharp excavator spoon or drilled with a round bur.
5. The pulp chamber was flushed with saline, and bleeding was stopped by gentle pressing with cotton ball. MTA (ProRoot, Densply, USA) was mixed with normal saline. The fracture surface of dental pulp was covered with prepared MTA, with a thickness of about 2 to 3 mm, and gently pressed with saline-containing cotton ball to be closely fitted with the radicular pulp.
6. The pulp chamber was filled with glass ionomer, and filled by composite resin for sealing (Filtek 250, 3M ESPE, USA).

### 7) PMC repair

The proximal-distal middle surface was cut, so that it was parallel to each other, or the tooth showed a slightly conical shape. The highly prominent position of buccal/lingual surface was ground to reduce the neck undercut. The line angle of intersection between the adjacent surface and the buccal/lingual surface should also be blunt and round. Afterwards, 1 mm was ground from the occlusal surface uniformly, the line angle with the axial surface should be blunt and round, and the dental neck could not have a shoulder. When the tooth had a short dental crown, it was prepared 0.5 mm away from the subgingival position. An appropriate PMC was chosen according to the tooth location and size after preparation, and normal occlusion was achieved.

### Pulpectomy group

1. All teeth were given block anesthesia with 2% hydrochloric acid lidocaine injection (5 mL: 0.1 g, Tianjin Jinyao Pharmaceutical Co., Ltd., China) or local infiltration anesthesia with primacaine injection (Acteon, France). Local anesthetics were chosen based on the voluntary principle. If block anesthesia using 2% hydrochloric acid lidocaine injection worked unsatisfactorily, local infiltration anesthesia was simultaneously conducted. Primacaine was chosen for local infiltration anesthesia in the vestibular groove on the buccal side.
2. The caries tissue was drilled using a sterile high-speed corundum ball to prepare a cavity, the pulp was opened, the roof of pulp chamber was removed, the crown pulp tissue was removed to find the root canal orifice, and the dental pulp was completely removed using a sterile nerve broach.
3. The pathological tissue and infected substances in the root canal were cleared. The root canal was flushed repeatedly with 2.5% sodium hypochlorite and normal saline, and the residual substances and debris were removed.
4. After being flushed and twisted dry with a sterile paper, the root canal was introduced or pressurized with filling material Vitapex (Morita, Japan) under moisture-proof conditions, the cavity lining was filled with glass ionomer, and filling was carried out for sealing. PMC repair and the remaining steps were the same as those for pulpotomy group.

### Clinical examination

Spontaneous pain, percussion pain, swelling or fistula of surrounding mucous membrane, and pathological and other abnormal loosening were examined and compared with those of normal teeth.

### X-ray examination

Periodontal ligament widening, shadows of root furcation and root tip, root canal calcification, and root canal absorptions were examined.

### Evaluation criteria

**N:** In clinical examination, normal molars had no pathological symptoms or signs like spontaneous pain, percussion pain, tenderness, swelling, fistula formation, or pathological loosening. There were no X-ray changes, such as periodontal ligament widening, shadows of root furcation and root tip, root canal calcification, and root canal absorptions.

**H:** In clinical examination, normal molars had no pathological symptoms or signs like spontaneous pain, percussion pain, tenderness, swelling, fistula formation, or pathological loosening. There were no X-ray changes compared with normal teeth in the same or contralateral jaw, except for physical absorption.

**P_0_:** Although there was pain history in clinical examination or periodontal ligament widening, root canal calcification and other pathological changes in X-ray examination, the teeth should not be removed immediately.

**P_x_:** The teeth which had all the above symptoms and signs in clinical and X-ray examinations should be removed immediately.

The results of N (or H or P_0_) in clinical and X-ray examinations were recorded as “effective”, and those of P_x_ were “ineffective”.

### Statistical analysis

All data were analyzed by SPSS20.0. The categorical data were expressed as (±SD), and inter-group comparisons were performed by the two independent samples t-test. The numerical data were expressed as percentage. P<0.05 was considered statistically significant.

## RESULTS

### Baseline clinical data

A total of 124 children (192 molars) with deep caries were included, with the mean age of (4.12 ± 1.08) years old. There were 58 boys and 66 girls. MTA pulpotomy was performed for 101 molars and Vitapex pulpectomy was conducted for 91 molars.

### Outcomes 18 months after surgery

As evidenced by clinical and imaging results 18 months after surgery, the proportion of molars without lesions was 80.20% in the pulpotomy group, which was significantly higher than that of the pulpectomy group (72.53%). The pulpotomy group with good clinical manifestations underwent spontaneous pain in four molars during follow-up, and five molars gradually underwent pain and gingival redness and swelling. The pulpectomy group suffered from occlusion discomfort in nine molars and gingival fistula in seven molars during follow-up. The postoperative morbidity of the pulpectomy group was significantly higher than that of the pulpotomy group (χ^2^=4.50, P=0.04) (Table-I).

**Table-I:** Outcomes 18 months after surgery (n/%).

	*N*	*H*	*P_0_*	*P_x_*	*Overall response rate*
Pulpotomy group (101)	81 (80.20)	6 (5.94)	4 (3.96)	10 (9.90)	91 (90.10)
Pulpectomy group (91)	66 (72.53)	5 (5.49)	1 (1.10)	19 (20.88)	72 (79.12)
P	<0.05				<0.05

### Pathological changes 18 months after surgery

There was no significant difference between the two groups in periodontal ligament widening, shadows of root furcation and root tip, root canal calcification or root canal absorptions (P>0.05) (Table-II).

**Table-II:** Pathological changes 18 months after surgery (n/%).

	*Periodontal ligament widening*	*Shadows of root furcation and root tip*	*Root canal calcification*	*Root canal absorptions*
Pulpotomy group (101)	16 (15.84)	16 (15.84)	3 (2.97)	12 (11.88)
Pulpectomy group (91)	18 (19.78)	18 (19.78)	0 (0)	15 (16.48)
P	>0.05	>0.05	>0.05	>0.05

### Tooth survival analysis

**Fig. 1:**
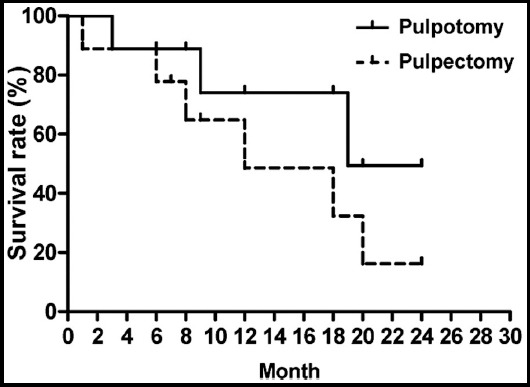
Tooth survival analysis.

Teeth did not survive if they were extracted due to pathological changes or underwent premature loss during follow-up. The 18-month tooth survival rates of pulpotomy and pulpectomy groups were 90% and 79% respectively, which were significantly different (χ^2^=4.645, P=0.031) (Fig.1).

## DISCUSSION

The chewing function of deciduous teeth has a great impact on the maxillofacial development, masticatory muscle exercise and function, normal eruption and arrangement of permanent teeth, children’s pronunciation, mental health and growth and development, and also is of great significance to the treatment and preservation of deciduous dental caries.^5^ Pulpotomy is to remove the pulp with pathological changes (coronal pulp or part of the root pulp)^6^, and retain the disease-free root pulp, with pulp capping covered on the root pulp section.^7^ The surface of the section was isolated from the external environment through new dentin, so that the teeth can maintain certain vitality.^8^ For the teeth with the root tip not formed yet, the root tip can still continue the growth and development until completion.^9^ Therefore, pulpotomy is a good method for biological treatment.^10^ The pulp is removed in pulpectomy under the guidance of the root canal instrument, and the root canal was initially dredged and expanded to clear the infection^11^, and temporarily filled with calcium hydroxide cataplasm with a highly effective bactericidal effect.^12^ With the assistance of the existing anesthesia technique and root canal measuring instrument^13^, the treatment can effectively control the pain^14^, and basically remove infected pulp, and does not fully expand the root canal during the operation^15^, with fewer risks of root canal offset and perforation and short treatment process, which is easy to be accepted by patients.^16^

In this study, 80.20% of the diseased teeth with pulpotomy had no clinical manifestations during the follow-up period, which accounted for 72.53% in pulpectomy. The pulpotomy group with good clinical manifestations underwent spontaneous pain in four molars during follow-up, and five molars gradually underwent pain and gingival redness and swelling. The pulpectomy group suffered from occlusion discomfort in nine molars and gingival fistula in seven molars during follow-up. The postoperative incidence of the pulpectomy group was significantly higher than that of the pulpotomy group. There were no significant differences in the periodontal ligament widening, root tip and root bifurcation shadow, root canal calcification and internal and external root canal absorption, but the results of the pulpotomy group were slightly lower than those of the pulpectomy group (P>0.05). The 18-month survival rate of pulpotomy and pulpectomy was 90% and 79%, respectively. Howley et al. performed FC pulpotomy and Vitapex filled pulpectomy on 100 cases of deciduous anterior teeth deep caries with pulp exposure in 29 children patients and followed them up for 24 months.^17^ The success rates of FC pulpotomy and Vitapex filled pulpectomy were 89% and 73%, respectively, without a significant difference. Aminabadi et al. conducted treatment for 100 cases of deciduous anterior teeth with deep caries and vital pulp with FC pulpotomy and pulpectomy filled with zinc oxide clove oil cement.^18^ Then 12- and 24-month follow-up observations showed that the clinical success rates of pulpotomy and pulpectomy were 86.9% and 95.6%, respectively, without a significant difference. At present, pulpotomy is recommended for diseased teeth in clinical treatment guidelines of the American Academy of Pediatric Dentistry (AAPD). Pulp treatment should preserve the vitality of the pulp, so as to maintain the integrity and health of the teeth and its support tissues. Part of the pulp can be retained through pulpotomy, so that the root can continue to develop to maintain the normal replacement of deciduous teeth with permanent teeth.

## CONCLUSION

The clinical effects of MTA pulpotomy and Vitapex pulpectomy under general anesthesia were significantly different. As suggested by the AAPD guidelines, the postoperative effects of pulpotomy are better than those of pulpectomy.

### Authors’ Contributions

**YT & WX:** Data collection and analysis.

**YT & WX:** study design and manuscript writing.
